# Effects of brewing conditions on infusible fluoride levels in tea and herbal products and probabilistic health risk assessment

**DOI:** 10.1038/s41598-021-93548-3

**Published:** 2021-07-08

**Authors:** Nattha Pattaravisitsate, Athit Phetrak, Thammanitchpol Denpetkul, Suthirat Kittipongvises, Keisuke Kuroda

**Affiliations:** 1 Research Office, Faculty of Dentistry, Mahidol University, Bangkok, Thailand; 2 Department of Social and Environmental Medicine, Faculty of Tropical Medicine, Mahidol University, Bangkok, Thailand; 3 Environmental Research Institute, Chulalongkorn University, Bangkok, Thailand; 4 Department of Environmental and Civil Engineering, Toyama Prefectural University, Imizu, Japan

**Keywords:** Environmental sciences, Risk factors

## Abstract

Excessive ingestion of fluorides might adversely affect the health of humans. Hence, this study aimed to investigate the concentrations of infusible fluoride in five different types of tea and herbal products; additionally, the probabilistic health risks associated with the ingestion of fluoride in drinking tea and herbal products were estimated. The highest and lowest concentrations of infusible fluoride were detected in black and white tea, respectively. On average, the highest amount of infusible fluoride was extracted following a short brewing time of 5 min in the case of black tea (2.54 mg/L), herbal tea (0.40 mg/L), and white tea (0.21 mg/L). The level of infusible fluoride during brewing was inversely associated with the leaf size of the tea and herbal products. Furthermore, the type of water used influenced the release of infusible fluoride; purified water yielded lower amounts of infused fluoride. The findings of the probabilistic health risk assessment indicated that the consumption of black tea can increase the fluoride intake leading to chronic exposure. Thus, the health risk posed by fluoride intake from drinking tea needs to be evaluated in more details in the future. Appropriate measures for health risk mitigation need to be implemented to minimize the total body burden of fluorides in humans.

## Introduction

Fluorine is the 13th most abundant element in the Earth’s crust (0.054% by weight) and is mostly present in the form of fluoride in various minerals. Fluoride is an essential micronutrient, and an appropriate amount of intake of this mineral, daily, can prevent the development of dental caries. However, numerous studies have indicated that excessive fluoride ingestion over a long period can lead to potentially severe dental and skeleton fluorosis, hypertension, damage to the neurological system, and a lower intelligence quotient^1–5^. Furthermore, it might have an adverse impact on the male reproductive system^6^.

Drinking water is the main source of fluoride intake in humans^7^. Approximately 90% of fluoride in drinking water can be absorbed in the human digestive system^7^. In Thailand, the guideline value of fluoride in drinking water is regulated at 0.7 mg/L, which is lower than that reported by the World Health Organization (WHO; < 1.5 mg/L)^8^. This was done to prevent the development of fluorosis in the country^9^. Nonetheless, the number of cases of dental fluorosis among children in Bangkok has been consistently increasing over the past two decades (18.4% as of 2014), possibly because of the intake of fluoride supplements^10^, thus indicating that the potential risk of excess fluoride consumption in Thailand might be a serious public health concern.

Tea (*Camellia sinensis*) is one of the most popular nonalcoholic beverages extensively consumed by health-conscious people in many societies^11,12^. The numerous types of teas available in the market could be broadly categorized into four types depending on the number of oxidation steps required during processing: black tea (fully oxidized), green tea (nonoxidized), oolong tea (semioxidized), and white tea (lightly oxidized)^13–15^. Herbal tea products, in which some contain the leaves of *Camellia sinensis*^11^, are also widely popular among consumers in Thailand and across the world^16^. The consumption of tea daily is increasing, and large amounts are consumed by adults (nearly 1 L per person per day on average) in oriental countries^11^. The positive health benefits of tea include the presence of nutrients and minerals, antioxidant activity, favorable taste, aroma, and anticancer properties^17–19^.

Tea contains a greater amount of fluoride than other plants, predominantly because it is an efficient fluoride accumulator^20^. Most of the fluoride in tea (nearly 98%) is accumulated in the leaves, particularly the mature leaves ^21^, and can be easily released during tea infusion^22,23^. Hence, the ingestion of fluoride from tea can lead to potential detrimental health effects. According to previous studies^13,22,24,25^, different types of tea, the brewing times, the tea leaf size, and the type of water used for tea infusion could influence the infusion of fluoride. However, the effects of these factors on releasable fluoride during infusion have not been evaluated in detail.

Therefore, the present study aimed to investigate the effects of different conditions of tea infusion, such as the type of tea used, the brewing time, the leaf size, and the type of water used, on the concentration of releasable fluoride from the infusion. Additionally, the associated probabilistic health risk following exposure to fluoride in drinking was evaluated using the Monte Carlo simulation.

## Materials and methods

### Collection and preparation of the tea and herbal products

Sixteen brands (locally made and imported) of tea and herbal products (five black tea, three green tea, three oolong tea, two white tea, and three herbal tea products) were purchased from a market in Bangkok, Thailand, in January 2019 (Table S1; as shown in supplementary information (SI)). Approximately 200 g of each tea and herbal product was collected and individually stored in airtight plastic bags (referred to as uncrushed tea samples). Approximately 100 g of each type of tea was crushed using an electrical grinder and individually packed into plastic bags. The ground samples were passed through a sieve (mesh size, 20) and stored in cleaned containers (hereafter referred to as crushed tea). The lengths of the tea leave in the uncrushed tea samples (*n* = 20) were carefully measured using a ruler. Dynamic light scattering was used to analyze the apparent hydrodynamic diameters of tiny tea particles ranging between 0.1 and 1000 µm in suspension (Partica mini LA-350, Horiba, Japan).

### Effect of tea infusion on fluoride release into the solution

Batch experiments of the infusion process were conducted using the one bottle point technique to investigate the levels of fluoride released from the tea and herbal products. The effects of the types of tea, brewing times (5, 10, and 20 min), leaf size (uncrushed and crushed tea samples), and types of water (distilled, ultrapure, water treated by reverse osmosis membrane system [RO], tap, bottled, and bottled mineral) were evaluated using a method similar to that described by Mahvi et al. (2006)^26^. Briefly, the dried tea sample (1.00 ± 0.02 g) was placed in a cotton bag, which was then placed in a beaker (100 mL) containing 50 mL of boiling water (2% by w/v). The prepared container was covered with a watch glass, and the tea was continuously brewed at ~ 100 °C for 5, 10, or 20 min. Subsequently, the tea bag was taken out, and the obtained solution was passed through a filter paper (pore size, 11 µm; Whatman No. 1, UK). A solution was prepared by adding water to a final volume of 50 mL, at room temperature. The total infusible fluoride concentration in the tea solution was quantified using a fluoride ion-selective electrode (VSTAR 40A, Orion, USA) as described previously by Kaophun et al. (2018)^27^. Each experiment was conducted in triplicate, and the average value ± the standard deviation (SD) was reported as the final result.

### Estimation of health risk

Health risk assessment is one of the methods used to indicate the possible harmful effects of environmental pollutant exposure on human health^28^. The equations used to evaluate the noncarcinogenic health risks of fluoride exposure from tea consumption in the present study are shown in Table 1^28^ among individuals belonging to different age groups (children, 0–10 years; teenagers, 11–20 years; and adults, 21–70 years)^22^. The probabilistic health risk assessment was conducted using Monte Carlo simulations with 10,000 iterations. Additionally, sensitivity analyses were conducted using the Oracle Crystal Ball software v.11.1.2.4.850 to determine the input parameters with the highest impact on the calculation of the probabilistic health risk assessment.

**Table 1:** Equations used to estimate the Human Exposure and Health Risk^28^.

Equations	Description
${\text{Chronic}} \cdot {\text{Daily}} \cdot {\text{Intake}} \cdot {\text{(CDI)}} = \frac{{{\text{C}} \times {\text{DI}} \times {\text{EF}} \times {\text{ED}}}}{{{\text{BW}} \times {\text{AT}}}}$	C is the fluoride concentration in tea infusion (mg/L) DI is the average daily intake rate of tea (L/day) EF is the exposure frequency (day(s)/year) ED is the exposure duration (year) BW is body weight (kg) AT is the averaging time (days) CDI is the chronic daily intake (mg/kg/day) RfD is the reference dose (mg/kg/day)
${\text{Hazard}} \cdot {\text{Quotient}} \cdot {\text{(HQ)}} = \frac{{{\text{CDI}}}}{{{\text{RfD}}}}$ An HQ of < 1 indicates an insignificant risk level, whereas an HQ of > 1 implies a potential noncancer-causing health impact	

### Statistical analysis, quality control, and quality assurance

The descriptive statistics were calculated using Microsoft Office Excel 2016. The normality of the data sets was tested using the Shapiro–Wilk test. Due to non-normal distribution of the data sets, the nonparametric Mann–Whitney U test was used for pair-wise comparisons using the SPSS statistical package (version 18.0). Spearman’s rank correlation analysis was used to investigate the correlations between the parameters (quality of water) used for infusion and infusible fluoride concentrations. A *p*-value of < 0.05 was considered significant.

### Analytical methods

The concentration of fluoride in water was analyzed using a fluoride ion-selective electrode (VSTAR 40A). The pH and electrical conductivity of the water samples were analyzed with a pH meter (Model 215, Denver Instrument) and conductivity meter (Model YSI-3200, YSI Incorporated, USA), respectively. The concentration of dissolved organic carbon (DOC) was determined using a total organic carbon (TOC) analyzer (TOC-L, Shimadzu, Japan) in the nonpurgeable organic matter mode. An ion chromatography (940 Professional IC Vario, Metrohm, Switzerland) was used to analyze the concentrations of the cations (such as sodium, potassium, calcium, and magnesium) and anions (such as chloride, bromide, nitrate, sulfate, and phosphate) in the water samples.

## Results and discussion

### Fluoride concentration in different types of tea and herbal products

**Figure 1:**
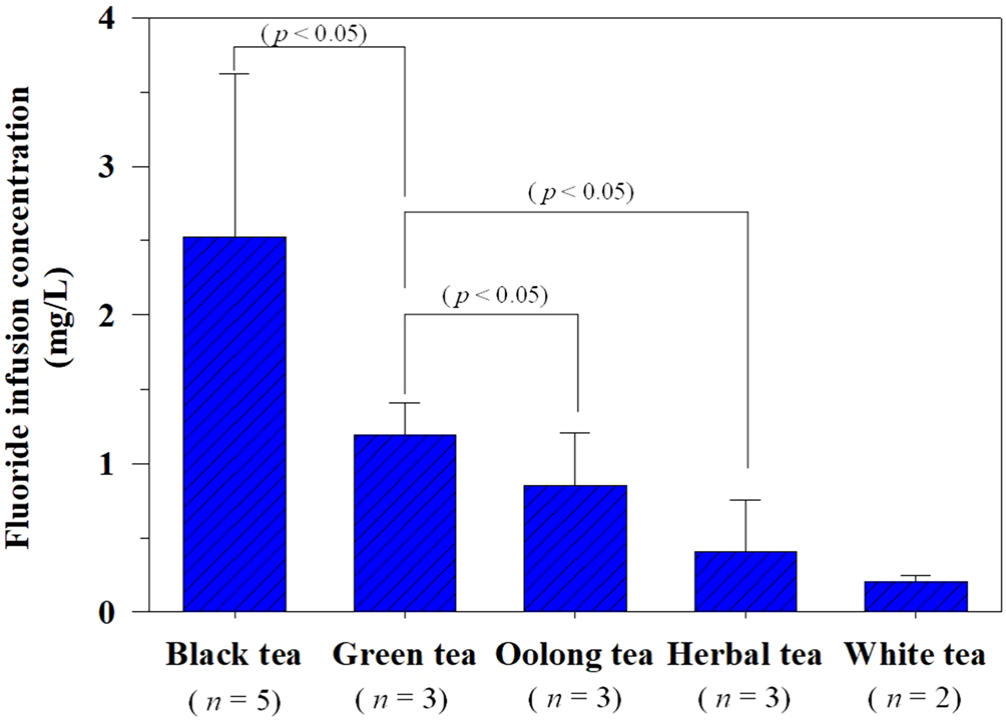
The content of fluoride in the various tea and herbal infusions after 5 min of brewing.

Tea is generally infused for a few minutes using hot water^11^. Therefore, a brewing time of 5 min for the infusion of the tea and herbal products was selected to evaluate the levels of infusible fluoride in the present study (Fig. 1). The amount of fluoride in the tea infusions was influenced by the type of tea used; the concentrations in descending order were as follows: black tea > green tea > Oolong tea > herbal tea > white tea. Among the tea products tested, black tea showed a high level of fluoride in the tea solution (average, 2.53 ± 1.10 mg/L; range, 1.12 ± 0.07–3.87 ± 0.06 mg/L), which is comparable with that (range, 0.32–4.54 mg/L) reported in the study by Malinowska et al. (2008)^29^. Likewise, other studies by Mahvi et al. (2006)^26^ and Emekli-Alturfan et al. (2009)^30^ reported high concentrations of infusible fluoride in black tea (2.60 and 3.72 mg/L, respectively).

The average concentration of infusible fluoride in green tea (1.19 ± 0.22 mg/L) was significantly lower than that of black tea (*p* < 0.05). The infusible fluoride content in green tea in this study was comparable with that (range, 0.16–3.29 mg/L) reported in the study by Maleki et al. (2016)^31^. The detection of lower fluoride levels in green tea infusion might be attributed to the method used for harvesting it (from the upper leaf bud and the next two leaves)^29,32^. Nonetheless, the fluoride levels were significantly greater than those observed in oolong tea (0.86 ± 0.35 mg/L; *p* = 0.006) and herbal tea (0.40 ± 0.35 mg/L; *p* = 0.005). The white tea infusion presented with the lowest fluoride concentration at 0.21 ± 0.04 mg/L (Fig. 1). This result is comparable with that reported by Malinowska et al. (2008)^29^, wherein the fluoride concentration in white tea liquor ranged from 0.37 to 0.54 mg/L. These differences in fluoride concentrations in the tea solutions might be due to the differences in the types of teas used, the harvesting seasons, and the soil conditions^22,29,33,34^. Furthermore, Cai et al. (2016)^21^ indicated that the content of fluoride was significantly correlated with the maturity of the tea leaves (*R*^*2*^ = 0.866; *p* < 0.05), which could be considered as another reason for the variations in fluoride concentrations in the present study.

### Fluoride concentration based on the brewing time

The effect of brewing time on infusible fluoride concentration was investigated, and the results are presented in Table 2. The mean fluoride concentrations of the tea and herbal infusions after 5, 10, and 20 min of brewing time were 1.28 ± 1.12, 1.33 ± 1.10, and 1.37 ± 1.09 mg/L, respectively. The amounts of fluoride in green tea and oolong tea infusion were found to increase with the increase in the brewing time from 5 to 20 min. The highest amount of infusible fluoride was detected in the green tea and oolong tea infusions at 20 min of brewing time (1.54 ± 0.44 mg/L and 0.99 ± 0.29 mg/L, respectively), indicating that the fluoride ions were continually extracted from these products during brewing. Thus, the brewing time could be considered as one of the significant factors that influence the release of fluoride from green tea and oolong tea infusions. These results are consistent with the findings reported in previous studies^22,29,35^. The extended brewing time might provide extended interactions between the tea particles and hot water during infusion, leading to a greater release of fluoride content in the solution.

**Table 2:** The concentration of fluoride from the different types of tea infusions after 5, 10, and 20 min of brewing time.

Type of product	Fluoride concentration					
	5 min		10 min		20 min	
	F-mg/L	SD	F-mg/L	SD	F-mg/L	SD
Black tea (*n* = 5)	2.54	1.10	2.55	0.95	2.55	0.99
Green tea (*n* = 3)	1.19	0.22	1.45	0.28	1.54	0.44
Oolong tea (*n* = 3)	0.86	0.35	0.91	0.45	0.99	0.29
Herbal tea (*n* = 3)	0.40	0.35	0.36	0.40	0.41	0.39
White tea (*n* = 2)	0.21	0.04	0.21	0.04	0.20	0.04
All products	1.28	1.12	1.33	1.10	1.37	1.09

Conversely, the levels of fluoride in the black tea, white tea, and herbal tea infusions remained relatively constant when the boiling time was extended from 5 to 20 min (Table 2), indicating that soluble fluoride can infuse into the solution within the short brewing time of 5 min. On the basis of these results, we presumed that brewing time might not influence the infusion of fluoride in black tea, white tea, and herbal tea infusions. Similar results in the case of herbal tea infusions have been reported in another study^30^. Additionally, in the study by Chan et al. (2013)^19^, soluble fluoride in a black blend infusion was mostly extracted within a brewing time of 2 min.

### Influence of leaf size on concentration of infusible fluoride during tea infusion

**Figure 2:**
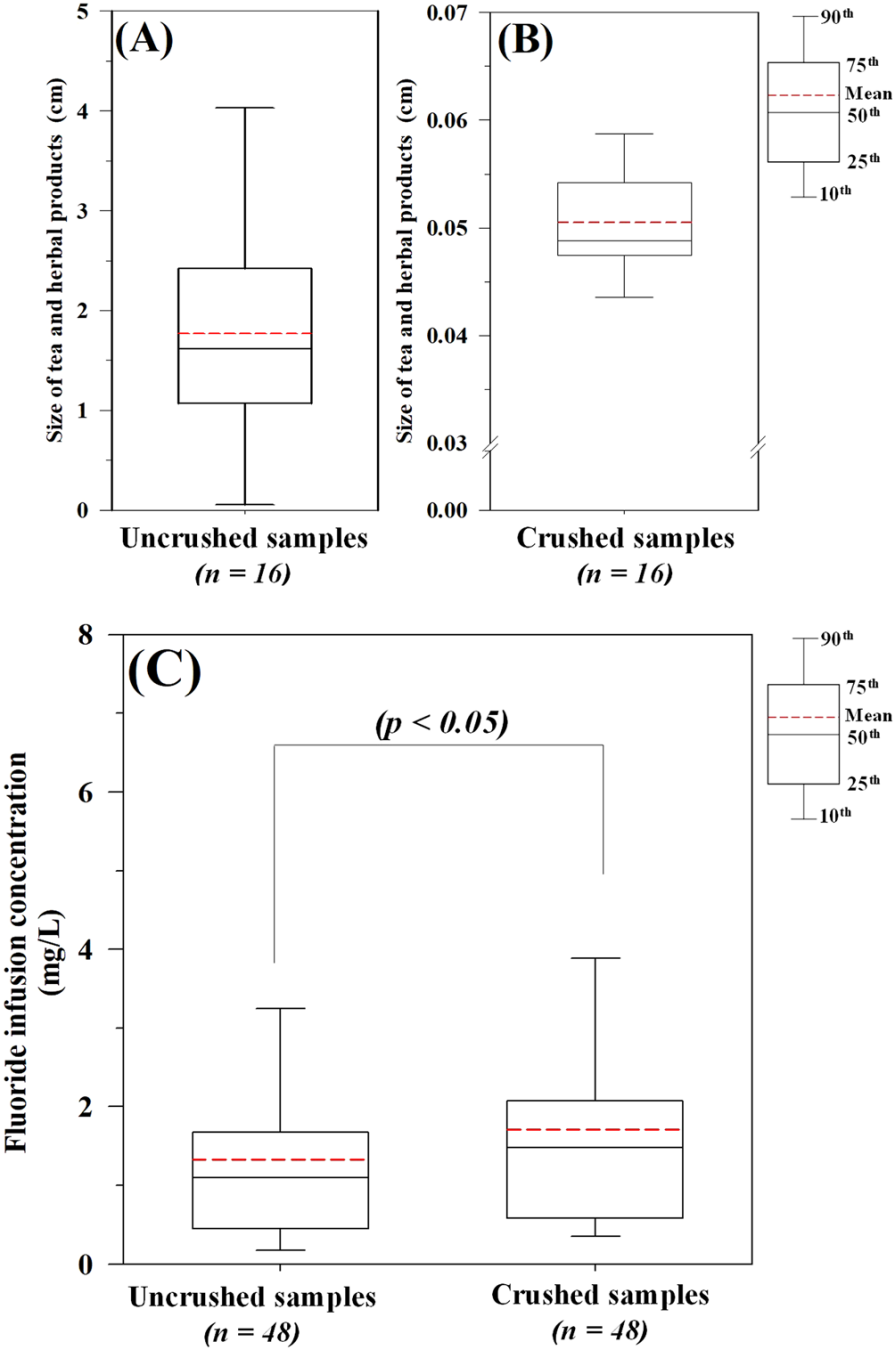
Influence of leaf size of tea and herbal products on fluoride concentration during infusion. Particle sizes of the (**A**) uncrushed and (**B**) crushed tea samples. (**C**) Comparison of infusible fluoride concentrations between the uncrushed and crushed tea infusions.

The size of the tea leaf is a significant parameter that can influence the release of fluoride during tea infusion^24^. Additionally, it is used as an important indicator of the quality of the tea product used during the fermentation process^36,37^. In the present study, high variations in leaf size were observed in the uncrushed tea samples (mean, 1.77 ± 1.22 cm; range, 0.06–4.11 cm; Fig. 2A). This might be due to the different methods used for processing the tea leaves. The particle sizes of the crushed tea samples were smaller at 0.04–0.06 cm (Fig. 2B).

The level of fluoride extract from the uncrushed tea infusion ranged between 0.02 and 3.87 mg/L (mean, 1.33 ± 1.10 mg/L; Fig. 2c). The highest concentration of infusible fluoride was detected in the black tea sample, whereas the herbal tea sample presented with the smallest amount of infusible fluoride. Alternatively, a relatively higher concentration of infusible fluoride was obtained from the crushed tea samples (mean concentration, 1.71 ± 1.36 mg/L; range, 0.03–5.39 mg/L). Thus, the mean concentrations of fluoride in the crushed tea infusions were significantly higher than those in the uncrushed tea infusions (*p* < 0.05) thereby indicating that the size of the tea leaf is an important parameter for fluoride release. Chan et al. (2013)^19^ reported that a change in the size of the tea particle affected the rate of infusion of fluoride; tea products with large particle size could have a slower rate of infusion because of the lower surface area when compared with those with small particle size.

### Influence of water sources on the release of fluoride during black tea infusion

**Figure 3:**
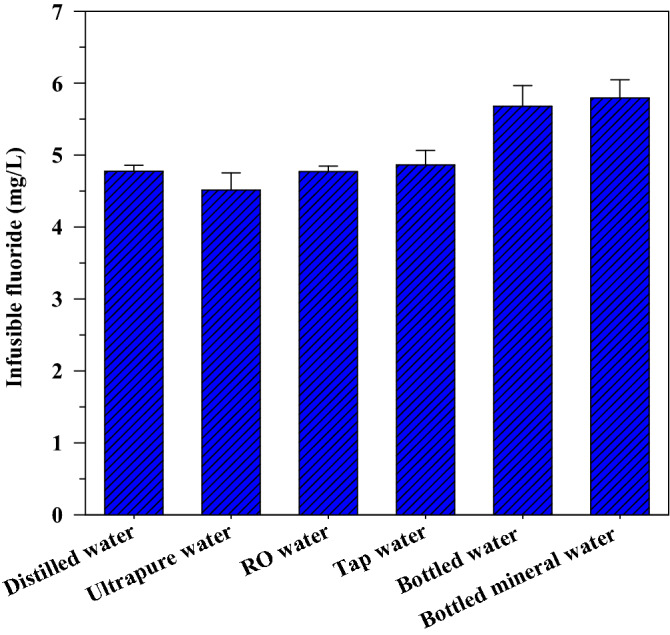
Effect of types of brewing water on fluoride infusion using crushed black tea with a brewing time of 5 min.

Water quality is an important factor that can influence the quality of the tea infusion^38,39^. Generally, tap water, bottled water, and purified water are used for tea infusion^13,25,40^. The highest level of fluoride infusion was observed during the brewing of the crushed black tea samples. Therefore, black tea was used to evaluate the effect of different types of water on the infusible fluoride concentration after a brewing time of 5 min (Fig. 3).

Table 3 shows the differences in the quality of the different types of water used for crushed black tea infusion. The initial fluoride concentration in bottled mineral water was 0.98 ± 0.02 mg/L, which was slightly higher than those in bottled water (0.51 ± 0.00 mg/L) and tap water (0.20 ± 0.01 mg/L). Nevertheless, the levels of fluoride in ultrapure water, RO water, and deionized water were lower than the detection limit in this analysis (< 0.02 mg/L). The pH values of tap water, bottled water, and bottled mineral water were within the recommended range for drinking water (6.5–8.5)^8^, whereas the rest of the water samples had pH values below 6.5. Both bottled water and bottled mineral water had a pH value of > 7, demonstrating a slightly alkaline pH. Tap water presented with the highest DOC concentration at 2.72 ± 0.17 mg/L. The concentration of most of the anions and cations in ultrapure water, RO water, and deionized water were markedly lower than those in tap water, bottled water, and bottled mineral water (Table 3); these findings were consistent with the results of the conductivity analysis and the finding of a previous study^13^. Ultrapure water, RO water, and deionized water are classified as purified water because of the low concentration of anions and cations and the low DOC levels^25^.

**Table 3:** Composition of the different ty of water used to prepare the tea infusion.

Parameter	Distilled water	Ultrapure water	RO water	Tap water	Bottled water	Bottled mineral water
pH	6.28 ± 0.12	5.94 ± 0.14	6.02 ± 0.01	7.11 ± 0.01	7.78 ± 0.03	8.02 ± 0.02
Conductivity (µS/Cm)	1.50 ± 0.71	2.50 ± 0.71	70 ± 1	1354 ± 3	642 ± 2	551 ± 1
Fluoride (mg/L)	< DL	< DL	< DL	0.176 ± 0.001	0.534 ± 0.003	1.084 ± 0.004
DOC (mg/L)	0.18 ± 0.08	0.30 ± 0.13	0.13 ± 0.02	2.72 ± 0.17	0.26 ± 0.10	0.20 ± 0.07
Sodium (mg/L)	0.261 ± 0.000	0.280 ± 0.000	8.091 ± 0.041	138.72 ± 0.04	113.39 ± 0.02	86.55 ± 0.08
Potassium (mg/L)	ND	0.015 ± 0.000	0.458 ± 0.005	7.616 ± 0.004	1.672 ± 0.006	0.976 ± 0.006
Calcium (mg/L)	ND	0.024 ± 0.000	1.285 ± 0.021	34.379 ± 0.005	3.006 ± 0.049	10.753 ± 0.026
Magnesium (mg/L)	0.031 ± 0.000	0.087 ± 0.000	0.547 ± 0.042	20.009 ± 0.046	0.714 ± 0.016	2.756 ± 0.002
Chloride (mg/L)	0.55 ± 0.01	0.63 ± 0.02	28.85 ± 0.06	326.75 ± 0.03	21.229 ± 0.03	6.390 ± 0.255
Bromide (mg/L)	ND	ND	0.032 ± 0.000	0.576 ± 0.000	0.077 ± 0.008	0.050 ± 0.001
Nitrate (mg/L)	ND	ND	0.640 ± 0.001	5.144 ± 0.000	0.469 ± 0.008	0.537 ± 0.009
Sulfate (mg/L)	0.24 ± 0.01	0.15 ± 0.01	1.74 ± 0.04	112.84 ± 0.01	39.46 ± 0.05	31.150 ± 0.010
Phosphate (mg/L)	ND	ND	ND	ND	ND	ND

The concentration of fluoride in distilled water, ultrapure water, and RO water was lower than the detection limit (DL) of 0.02 mg/L. ND, not detected.

The concentrations of infusible fluoride in crushed black tea infusions varied with the type of water used (Fig. 3). The mean concentrations of fluoride in the crushed black tea infusions prepared by bottled mineral water (5.79 ± 0.25 mg/L) and bottled water (5.68 ± 0.29 mg/L) were greater than those prepared by distilled water (4.78 ± 0.08 mg/L), ultrapure water (4.51 ± 0.24 mg/L), RO water (4.77 ± 0.08 mg/L), and tap water (4.86 ± 0.02 mg/L). These findings indicated that the type of water used for preparing a tea infusion might influence the leaching of fluoride and were consistent with those reported by Chandrajith et al. (2007)^36^ and Danrong et al. (2009)^41^. Furthermore, positive correlations were observed between infusible and initial fluoride concentrations (rho = 1; *p* < 0.01) and the initial pH value (rho = 0.941; *p* < 0.01) in the present study (Table S2; as shown in SI). This implies that both the initial fluoride concentration and the pH value of the water used for tea infusion might contribute to an increase in the concentration of the infusible fluoride. Some studies have reported that tea infusions made with water containing high concentrations of fluoride contributed to lower levels of infusible fluoride in the tea solution because tea leaves can absorb infusible fluoride ions^23,36^. The contradictory findings in the present study might be explained by the fact that increased levels of bicarbonate and hydroxide ions (alkaline pH) in bottled and bottled mineral water compete for fluoride adsorption by the tea leaves^42^ resulting in higher levels of infusible fluoride in the tea solution. Although fluoride is essential for human health, excessive fluoride intake can cause skeletal and dental fluorosis^5,43^. Thus, based on the findings of this study, it is recommended to use purified water for tea infusion to minimize fluoride exposure.

### Probabilistic risk assessment of fluoride in the different tea brands

Health risk assessment using a probabilistic approach with the Monte Carlo simulation, was used to evaluate the adverse health effects of toxic chemicals to obtain a more realistic risk estimation as compared with that obtained using the traditional deterministic risk assessment approach^44^. The estimated risk value with a probability distribution was achieved by this method resulting in its use to assess the potential health risk of pollutants in various environmental media^21,45^.

The health risk assessment from fluoride exposure through the consumption of different types of tea and herbal products was analyzed using the Monte Carlo simulations. The chronic daily intake (CDI) was calculated to estimate the nonadverse health effect of fluoride exposure from tea. The concentrations of infusible fluoride obtained after the different brewing times for each type of tea and herbal product were utilized and fitted with triangular distribution using Oracle Crystal Ball (Table S3; as shown in SI). The different exposure parameters, such as daily intake (DI) of tea, exposure frequency (EF), exposure duration (ED), body weight (BW), average time and RfD, were selected from previous studies, and their probability distributions among age groups are displayed in Table S4 (as shown in SI). It is noteworthy that the estimation of health risk for ingestion of fluoride in drinking tea and herbal products was calculated based on the input parameters shown in Tables S3 and S4 (as shown in SI).

**Figure 4:**
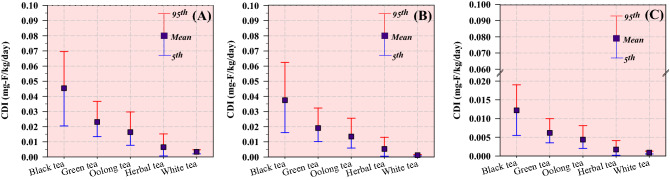
CDI in the (**A**) children, (**B**) teenagers, and (**C**) adults for the consumption of uncrushed tea and herbal products.

Figure 4 shows the output of the estimated CDI values with different percentiles (5th, mean, and 95th) from the various tea and herbal products in the different age groups. USEPA estimated the reference dose (RfD) as the maximum permissible risk for daily exposure (dental fluorosis in children and for skeletal fluorosis in adults at 0.06 and 0.12 mg-F/kg/day, respectively^46^. In the present study, the trend of estimated CDI values for black tea was relatively higher than those for the other tea products among the studied groups. The 95th percentile of the CDI values for black tea was greater than 0.06 mg-F/kg/day, particularly in children and teens. Alternatively, the other products presented with lower estimated CDI values, which remained below the aforementioned limit for dental fluorosis in all age groups. The results of the present study were comparable with those reported by Miri et al. (2018)^22^, where CDI values higher than 0.06 mg-F/mg/day (CDI at 95th percentile, 0.138 mg-F/kg/day) were detected in children who consumed Iranian tea, Kenyan tea (CDI at 95th percentile, 0.112 mg-F/kg/day), and Taksetare tea (CDI at 95th percentile, 0.066 mg-F/kg/day). Children could be considered as a vulnerable group at a higher risk of fluoride ingestion because of their body size^47^. Thus, the findings of the present study indicate that black tea has higher levels of infusible fluoride and could lead to dental fluorosis in children.

**Figure 5:**
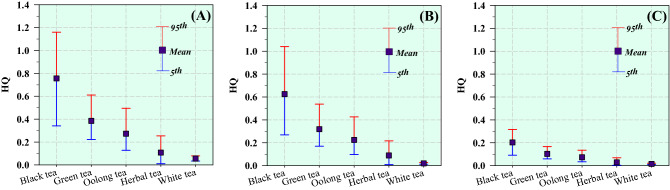
HQ values in (**A**) children, (**B**) teenagers, and (**C**) adults for the consumption of uncrushed tea and herbal products.

Figure 5 shows the calculated HQ values in the different age groups after the consumption of the different tea and herbal products. The potential adverse health hazards of fluoride exposure to people can be quantified when the HQ value exceeds 1.00^22^. In the present study, the HQ values for green tea, herbal tea, oolong tea, and white tea were below 1.00 in all the age groups, whereas the 95th percentile of the forecasted HQ values for black tea were 1.16 and 1.04 among children and teens, respectively. These results suggest that the ingestion of fluoride from drinking black tea over a long period can pose a potential noncarcinogenic health risk to children and teens. Furthermore, increased black tea consumption might increase the noncarcinogenic risk level because of the high amounts of fluoride ingested^30^. A higher predicted HQ value for black tea was observed among children in this study, implying that they were at a higher risk of fluoride ingestion. Thus, it is recommended that health education in school should be promoted to raise the awareness and understanding of fluorosis among children. Additionally, tea manufacturers should provide nutritional information regarding the concentration of fluoride on the packages of tea and herbal products to create awareness and minimize excess fluoride intake.

#### Sensitivity and uncertainty analyses

The sensitivity analysis was conducted to identify the parameter that contributed the most to the risk assessment. Figure S1 (as shown in SI) illustrates the results of the analysis for the noncarcinogenic risk assessment of fluoride exposure from tea among the various age groups in this study. The two main input parameters that contributed to the variances in the noncancer health risks in children were fluoride concentration (52%–96%) and frequency of exposure (3%–32%). BW had a barely negative impact on risk estimation in children with a contribution to variance ranging from − 0.5 to − 12.5%.

Fluoride concentration had the highest influence on the risk estimation with a contribution to variance ranging from 53 to 92% in teens and 48% to 95% in adults. Furthermore, the contribution to variance for exposure frequency and DI respectively ranged 3%–37% and 5%–31% in teens and 3%–30% and 1%–21% in adults. Hence, based on these results, the concentration of fluoride in tea could be the most important variable in estimating the health risk among the age groups evaluated in this study because of its high sensitivity (approximately > 50%).

To quantify the uncertainties during risk evaluation using the Monte Caro simulations, other input variables during risk assessment, especially those parameters identified by the sensitivity analysis, were reconsidered. The experiments were conducted in triplicate, and the concentrations of fluoride in the solution were analyzed using appropriate methods to minimize the uncertainties and increase the accuracy of the measurement. Furthermore, the obtained parameters from the previous published literature, such as exposure frequency and DI, may cause the uncertainties in this risk estimation. For instance, the consumption rate of tea might vary depending on the climatic conditions^48^. Moreover, personal lifestyle habits and cultural conditions might affect the daily ingestion of fluoride^45,48^. Additional studies focusing on these parameters are required to enhance the accuracy of the risk estimation among tea consumers in Thailand. Fluoride intake could be increased from other sources, such as drinking water, bottled mineral water, food, beverages, and fluoride supplements^33,43^. Therefore, the risk estimation in the present study could be underestimated because these other sources of fluoride were not included. Additional data regarding the different sources of fluoride should be included in future studies.

## Conclusions

Excessive ingestion of fluorides can prove detrimental to human health. In the present study, the effects of various brewing conditions on infusible fluoride were investigated in different types of tea and herbal products. The concentration of infusible fluoride in the tea infusion was significantly associated with the type of product used. The highest fluoride concentration was detected in black tea infusion, followed by green tea, oolong tea, herbal tea, and white tea infusions. The soluble fluoride in black tea, white tea, and herbal tea was mostly released within a short brewing time of 5 min. A decrease in leaf size significantly affected the amount of infusible fluoride in infusion. Likewise, the type of water used for brewing proved to be an important factor in lowering the amount of infusible fluoride. Furthermore, the 95th percentile of the estimated HQ values for black tea exceeded 1 among children and teens, indicating that these groups were at increased risk of experiencing the noncarcinogenic effects of excessive fluoride ingestion from black tea. Additional studies are required to evaluate the health risks posed by fluoride intake from drinking tea and to implement appropriate measures to mitigate these risks to minimize the total body burden of fluorides in humans.

## Supplementary Information

Supplementary Information.

## Data Availability

Supplementary data associated with this article can be found in the online version.
