# Supplementary material for: Effects of brewing conditions on infusible fluoride levels in tea and herbal products and probabilistic health risk assessment
Source: Sci Rep. 2021 Jul 8;11:14115. doi: 10.1038/s41598-021-93548-3 (PMC8266875; doi:10.1038/s41598-021-93548-3)
Supplement: Supplementary file 1 — Supplementary Information. [file 41598_2021_93548_MOESM1_ESM.pdf]

Supplementary information

Effects of brewing conditions on infusible fluoride levels in tea and herbal products and probabilistic health risk assessment

Nattha Pattaravisitsate[a], Athit Phetrak[b, *], Thammanitchpol Denpetkul[b], Suthirat Kittipongvises[c], Keisuke Kuroda[d]

[a]Research office, Faculty of Dentistry, Mahidol University, Bangkok, Thailand.

[b]Department of Social and Environmental Medicine, Faculty of Tropical Medicine, Mahidol University, Bangkok, Thailand.

[c]Environmental Research Institute, Chulalongkorn University, Bangkok, Thailand.

[d]Department of Environmental and Civil Engineering, Toyama Prefectural University, Imizu, Japan.

*Corresponding author: Athit Phetrak Ph.D

E-mail: athit.phe@mahidol.ac.th; athit.phetrak@gmail.com

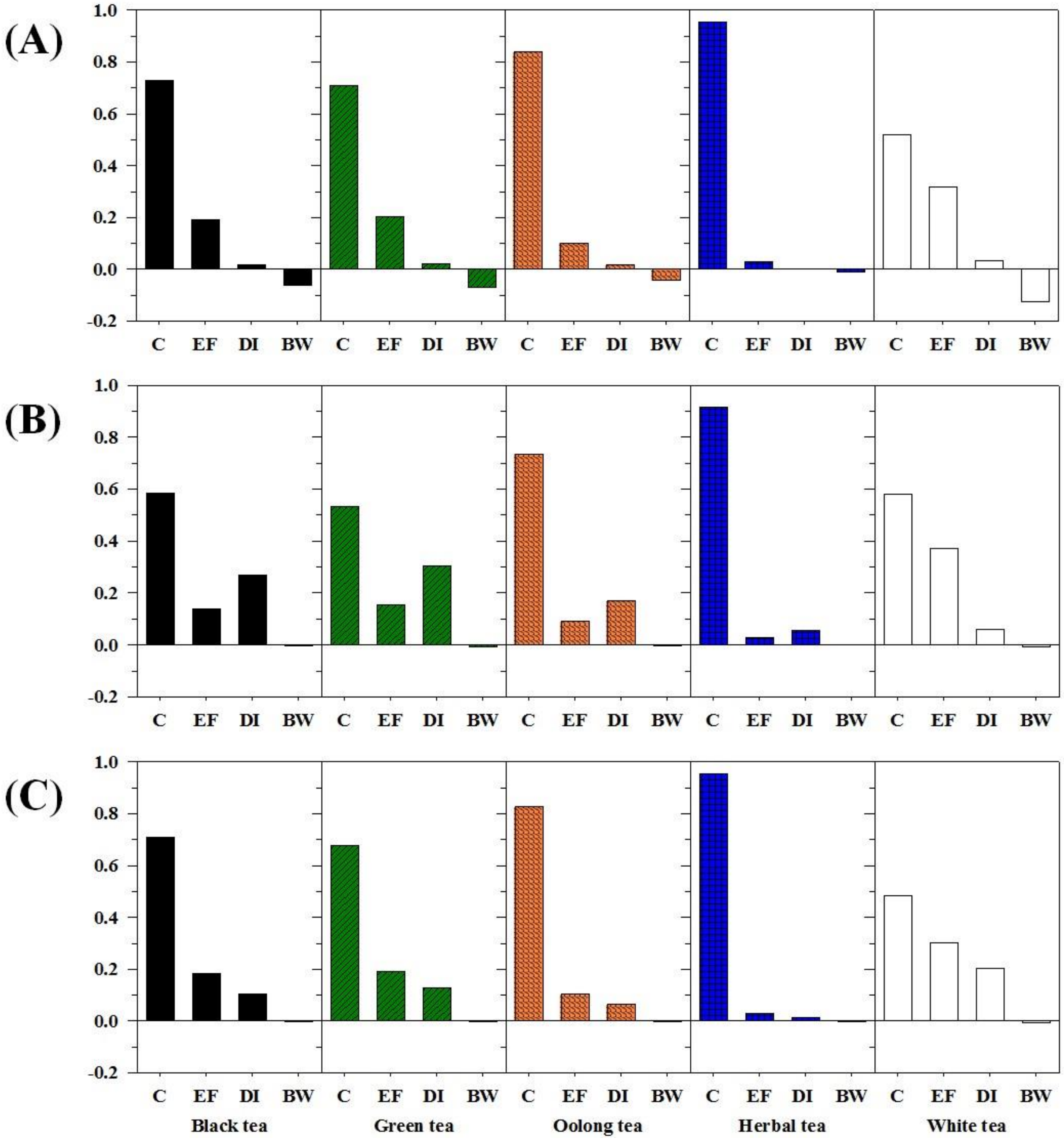

Figure S1: Sensitivity analysis of hazard quotient for fluoride exposure of different tea and herbal products; (A) in children, (B) in teens and (C) in adults.

Note: C is fluoride concentration in tea infusion, EF is exposure frequency, DI is the average daily intake rate of tea and BW is body weight.

Table S1 Tea and herbal products used for tea infusion in the study.

Type and brand	Country of marketing	Shape	Tea type and ingredients
**Green tea**			
1.Thai green tea	Thailand	Needle-like	Natural tea leaves
2.Thai green tea	Thailand	Needle-like	Green tea leaves
3.Thai green tea	Thailand	Compressed ball	Green tea leaves
**Oolong tea**			
1.Thai oolong tea	Thailand	Compressed ball	Natural tea leaves
2.Thai oolong tea	Thailand	Compressed ball	Natural tea leaves
3.Thai oolong tea	Thailand	Compressed ball	Natural tea leaves
**Black tea**			
1.Thai Black tea	Thailand	Needle-like	Black tea
2.Thai Black tea	Thailand	Needle-like	Black tea
3.Black tea	UK	Small granule	Black tea with a citrus bergamot flavor
4.Black tea	Indonesia	Small granule	Black tea
5.Thai Black tea	Thailand	Small granule	Tea powder with sugar, artificial flavor, artificial color
**White tea**			
1.Thai white tea	Thailand	Needle-like	Dried Flower tea
2.Thai white tea	Thailand	Needle-like	-
**Herbal tea**			
1.Jasmin tea	Thailand	Leaf-like	Green tea with jasmine
2.Mulberry tea	Thailand	Leaf-like	flower
3.Gynostemma pentaphyllum tea	Thailand	Small Leaf-like	Mulberry Gynostemma pentaphyllum

Table S2 Spearman correlations between the parameters (quality of water) used for infusion and infusible fluoride concentrations (n=6)

	F_infused	pH	Conductivity	DOC	Na	K	Ca	Mg	Cl	Br	Nitrate	Sulfate	F_initial
**F_infused**	1.000												
**pH**	1.000**	1.000											
**Conductivity**	0.600	0.600	1.000										
**DOC**	0.116	0.116	0.5220	1.000									
**Na**	0.600	0.600	1.000**	0.522	1.000								
**K**	0.600	0.600	1.000**	0.522	1.000**	1.000							
**Ca**	0.657	0.657	0.943**	0.522	0.943**	0.943**	1.000						
**Mg**	0.657	0.657	0.943**	0.522	0.943**	0.943**	1.000**	1.000					
**Cl**	0.200	0.200	0.829*	0.232	0.829*	0.829*	0.771	0.771	1.000				
**Br**	0.203	0.203	0.406	0.294	0.406	0.406	0.638	0.638	0.522	1.000			
**Nitrate**	0.319	0.319	0.754	0.147	0.754	0.754	0.812*	0.812*	0.928**	0.765	1.000		
**Sulfate**	0.714	0.714	0.943**	0.348	0.943**	0.943**	0.886*	0.886*	0.771	0.406	0.754	1.000	
**F_initial**	0.941**	0.941**	0.698	0.308	0.698	0.698	0.759	0.759	0.273	0.216	0.339	0.698	1.000

**. Correlation is significant at the 0.01 level.
*. Correlation is significant at the 0.05 level.

Table S3: Fluoride concentration distributions in each tea group

Type of tea	Fluoride concentration Unit	Normal tea	
		N	Distribution
Black tea	mg/L	45	Triangular (0.601, 3.940, 3.972) *
Green tea	mg/L	27	Triangular (0.911, 0.935, 2.505) *
Oolong tea	mg/L	27	Triangular (0.462, 0.487, 2.139) *
White tea	mg/L	18	Triangular (0.107, 0.260, 0.263) *
Herbal tea	mg/L	27	Triangular (0.004, 0.021, 1.183) *

Note: *Triangular (minimum, likeliest, maximum)

Table S4: Exposure parameters of the probabilistic risk model

Input parameter	Unit	Distribution	Reference
Daily Intake (DI)	L/day	Children: Uniform (0.3,0.5) *<br>Teens; Uniform (0.5,1) *<br>Adults: Uniform (1,1.5) *	Miri et al. (2018)
Exposure Frequency (EF)	Day/year	Triangular (180,345,365) **	Smith (1994)
Exposure Duration (ED)	Year	Fixed value (6)	Huang et al. (2017)
Body weight (BW)	Kg	Children: Lognormal (16.68,1.48)***<br>Teens: Lognormal (46.25,1.18)***<br>Adults: Lognormal (57.03,1.10)***	Wu et al. (2011)
Average Time (AT)	Day	Children: Fixed value (2190)<br>Teens: Fixed value (2190)<br>Adults: Fixed value (9125)	Huang et al. (2017)
Oral Reference Dose (RfD)	mg-F/kg.day	Fixed value (0.06)	Fallahadeh et al. (2018)

Note:
* Uniform (minimum, maximum)
**Triangular (minimum, likeliest, maximum)
*** Lognormal (Geometric mean, geometric standard deviation)

References:

Fallahzadeh RA, Miri M, Taghavi M, Gholizadeh A, Anbarani R, Hosseini-Bandegharaei A, Ferrante M, Conti GO (2018) Spatial variation and probabilistic risk assessment of exposure to fluoride in drinking water. Food Chem Toxicol. 113:314-321. https://doi.org/10.1016/j.fct.2018.02.001

Huang D, Yang J, Wei X, Qin J, Ou S, Zhang Z, Zou Y (2017) Probabilistic risk assessment of Chinese residents' exposure to fluoride in improved drinking water in endemic fluorosis areas. Environ Pollut. 222:118-125. https://doi.org/10.1016/j.envpol.2016.12.074

Miri M, Bhatnagar A, Mahdavi Y, Basiri L, Nakhaei A, Khosravi R, Eslami H, Ghasemi SM, Balarak D, Alizadeh A, Mohammadi A (2018) Probabilistic risk assessment of exposure to fluoride in most consumed brands of tea in the Middle East. Food Chem Toxicol. 115:267-272. https://doi.org/10.1016/j.fct.2018.03.023

Smith RL (1994) Use of Monte Carlo simulation for human exposure assessment at a superfund site. Risk Anal. 14:433-439. https://doi.org/10.1111/j.1539-6924.1994.tb00261.x

Wu B, Zhang Y, Zhang XX, Cheng SP (2011) Health risk assessment of polycyclic aromatic hydrocarbons in the source water and drinking water of China: quantitative analysis based on published monitoring data. Sci Total Environ. 410:112-118. https://doi.org/10.1016/j.scitotenv.2011.09.046
